# A cool hybrid approach to the herpesvirus ‘life’ cycle^☆^

**DOI:** 10.1016/j.coviro.2014.01.008

**Published:** 2014-04

**Authors:** Tzviya Zeev-Ben-Mordehai, Christoph Hagen, Kay Grünewald

**Affiliations:** Oxford Particle Imaging Centre, Division of Structural Biology, Wellcome Trust Centre for Human Genetics, University of Oxford, Oxford OX3 7BN, UK

## Abstract

Electron cryo tomography (cryoET) is an ideal technique to study virus–host interactions at molecular resolution. Imaging of biological specimens in a frozen-hydrated state assures a close to native environment. Various virus–host cell interactions have been analysed in this way, with the herpesvirus ‘life’ cycle being the most comprehensively studied. The data obtained were further integrated with fluorescence and soft X-ray cryo microscopy data applied on experimental systems covering a wide range of biological complexity. This hybrid approach combines dynamic with static imaging and spans a resolution range from micrometres to angstroms. Along selected aspects of the herpesvirus replication cycle, we describe dedicated combinations of approaches and how subsequent data integration enables insights towards a functional understanding of the underlying processes.

## Highlights

• Studying virus ‘life’ cycle in native conditions requires a hybrid approach.
• Fluorescence, X-ray and electron cryo microscopy combines dynamic with static imaging.
• This covers biological complexity with resolution from micrometres to angstroms.
• Integration of data provides insightful understanding of herpesvirus replication.
• Outlook: developments in sample thinning, instrumentation and computational analysis.

## Graphical abstract

**Figure:**
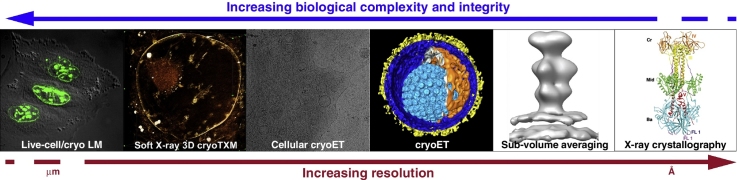

**Current Opinion in Virology** 2014, **5**:42–49

This review comes from a themed issue on **Virus structure and function**

Edited by **Wah Chiu, Thibaut Crépin** and **Rob WH Ruigrok**

For a complete overview see the Issue and the Editorial

Available online 16th February 2014

1879-6257/$ – see front matter, © 2014 The Authors. Published by Elsevier B.V. All rights reserved.

**http://dx.doi.org/10.1016/j.coviro.2014.01.008**

## Introduction

**Figure 1:**
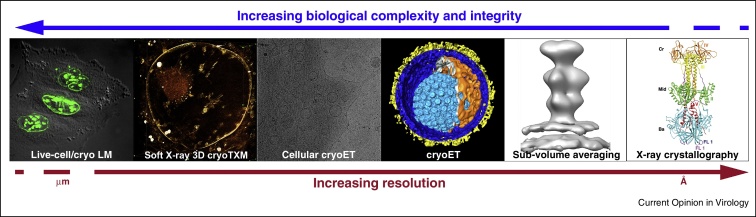
The spectrum of techniques applied to study the herpesvirus ‘life’ cycle. An integrated approach combining high-resolution structure determination methods and correlative light, soft X-ray cryo and electron cryo microscopy allows looking at dynamic processes at different resolution and complexity and ultimately leads to a better perception of those processes. LM, light microscopy; cryoTXM, transmission X-ray cryo microscopy; cryoET, electron cryo tomography.

Understanding virus ‘life’ cycles has a great impact on basic cell biological research as well as on the development of specific interventions and therapeutics. Accordingly, virus-host interactions have been the subject of many studies. Much of the structural data published comes from classical electron microscopy (EM) and tomography methods that involve fixation, dehydration, staining, and plastic embedding of the specimen [1–4]. However, interpretation of the results from these methods is limited, as the harsh sample preparation frequently leads to structural impairment of the biological specimens [2,5]. In the past decade, a variety of dedicated techniques have been developed to study viral replication cycles under more native conditions. Each technique has its strengths and weaknesses, often reflected as a compromise between the biological complexity covered and the achievable resolution (Figure 1). Thus, only the integration of the data obtained from all the techniques can provide the detail and context to understand the complex biological processes in a viral replication cycle.

EM is still in the centre of this spectrum of techniques (Figure 1), between higher resolution methods like X-ray crystallography and lower resolution light/fluorescence microscopy techniques that provide access to dynamic information of the concerned processes. However, classical EM is increasingly being replaced with electron cryo microscopy (cryoEM). The essence of cryoEM is visualizing biological specimens embedded in a thin film of vitreous ice (non-crystalline, amorphous, glass-like), the thickness of which is only slightly greater than the diameter of the specimen [6]. Imaging in the frozen-hydrated state, that is, keeping the water, preserves the genuine environment for the biological specimen. The complexity of the environment can range from macromolecules in buffer solutions (for recent examples see [7,8]) to intact cells [9,10] and tissues [11]. The limitation of cryoEM lay in the amenable thickness of the specimens to be imaged and in the achievable resolution. Soft X-ray cryo microscopy, particularly when performed in correlation with fluorescence microscopy, is an exciting emerging technique allowing the visualization of thicker and larger specimen areas and thus significantly complements cryoEM [12–14,15^•^]. On the higher resolution end, constant developments in computational analysis of cryoEM data hold promise for the future. In the most favourable cases it already provided atomic resolution information [8,16], and commonly produces molecular resolution that allows fitting of high-resolution crystal structures.

The ability of electron cryo tomography (cryoET) to visualize unique biological events in 3D makes its application to imaging macromolecules in their cellular and subcellular context very attractive [17]. In cryoET, a tilt series of projection images is collected and then combined computationally to reconstruct a 3D density map. Following the first studies of pleomorphic viruses by cryoET (reviewed in [18]), studying virus–host interactions by cellular cryoET has given unprecedented snapshots of the molecular interactions in the course of virus infection and replication in its host cell [19–24]. More recently, fluorescent microscopy has been integrated into cryoEM to help identify sites of interest, an approach referred to as correlative microscopy [25].

Here we describe how this spectrum of techniques and hybrid approaches have advanced our understanding of the herpesvirus replication cycle, as it is one of the most comprehensively studied examples of these techniques to date.

## The herpes simplex virus ‘life’ cycle

Herpes simplex virus type 1 (HSV1) is a highly ubiquitous human pathogen. It is the major cause of cold sores and, much more rarely, of fatal encephalitis. It is the prototypic species of the subfamily *Alphaherpesvirinae* from the larger *Herpesviridae* family of animal pathogens. Viruses in this family are comprised of large enveloped DNA viruses of complex structure [26]. A lipid bilayer envelopes an icosahedral capsid that in turn encapsulates a linear, double-stranded DNA genome. The lipid envelope is separated from the capsid by a proteinaceous matrix called the tegument [27]. The HSV1 virion 3D structure was the first pleomorphic enveloped virus structure to be determined by cryoET [28]. The 3D structure revealed that the ∼220-nm-diameter virions are bipolar, with the capsid being positioned eccentrically, thus forming a proximal and a distal pole. The envelope membrane is highly studded with viral glycoproteins with a non-random distribution, viz., being more abundant around the capsid distal pole, with implications for viral entry and assembly (see below).

**Figure 2:**
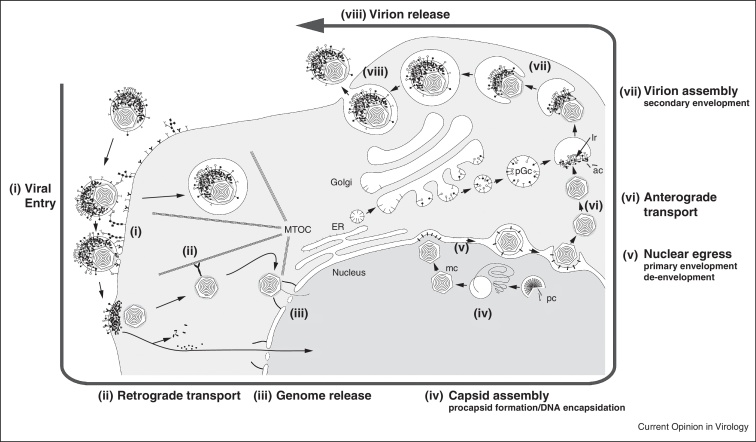
Illustration of the herpesvirus ‘life’ cycle. Virus infection starts with cell entry that involves the fusion of the viral envelope with that of the host, either directly at the plasma membrane or in an endosome leading to release of the capsid into the cytosol (i) [40]. The capsid is then transported retrogradely to the nucleus along microtubules (ii) [58]. The viral genome is released into the nucleus through the nuclear pore (iii) [59]. Transcription of viral genes and genome replication occur in the nucleus, as well as procapsid (pc) formation and DNA encapsidation (iv) [30]. Capsids exit the nucleus by a primary envelopment and de-envelopment mechanism (v) [43]. Capsids are then transported anterogradely to the point of virion assembly (vi). Virion assembly, including secondary envelopment and tegumentation, occurs close to the cell surface by budding into cellular vesicles originating from the Golgi that contain the viral glycoproteins on the lumenal side and accrete tegument proteins on the cytosolic (vii). Virions are released from the cell by fusion of the cellular vesicles with the plasma membrane (viii).

The complexity of the virions translates to a complex infection cycle (Figure 2). HSV1 replicates in epithelial cells that are the point of first entry, but gets transported retrogradely to sensory neurons where it establishes latent infection [29]. Virus replication takes place in two separated cellular compartments. Procapsid formation and packaging of the viral genome takes place at the nucleus [30], while tegumentation and envelopment proceeds in the cytoplasm [31]. As such, the HSV1 replication cycle spans the whole cell and often takes advantage of available cellular machineries for replication.

## Herpesvirus entry into host cells

**Figure 3:**
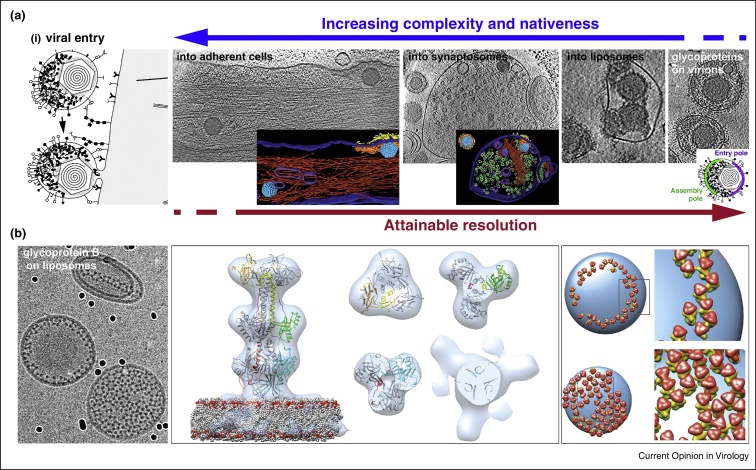
Dedicated experimental systems used for the study of HSV1 entry. **(a)** Reducing biological complexity from the most native system, virus entry into intact host cells [24], to gradually less complex subsystems, that is, virus entry into synaptosomes [24] or liposomes, and study of glycoproteins on the viral surface. **(b)** HSV1 glycoprotein B (gB), a key component of the fusion machinery, bound to liposomes used as display platform for direct visualization of fusion protein interaction with its target membrane. Sub-volume averaging was used to reconstruct the gB–lipid bilayer complex (middle panel, light grey) [42]. The EM reconstruction together with fitting of the gB crystal structure revealed the mode of interaction to the membrane. Lateral interaction of gB induced protein coat or belt formation on liposomes. Placing back the EM reconstruction in the experimentally determined orientations (right panel) allowed analysing the lateral interactions [42].

Herpesvirus entry requires fusion of the viral membrane with that of the host. Depending on the cell type, entry can take place at either the plasma membrane or out of the endosome after endocytosis [32,33]. Entry of HSV1 into flat adherent cells was imaged by cryoET and provided 3D snapshots of native fusion intermediates at the plasma membrane [24] (Figure 3a). Post-fusion, capsids released into the cytosol were detected between actin bundles very early after infection. Clusters of glycoprotein spikes protruding from the plasma membrane marked the entry sites. The majority of the tegument formed a thick layer just underneath the plasma membrane, clearly corresponding in shape and extent to the patch of glycoprotein spikes on the outer face of the plasma membrane. Specimen thickness is the main limiting factor in cellular cryoET. The electron beam penetration limit is about 0.5–1 μm and the achievable resolution depends inversely on the specimen thickness [34]. Though many organelles and subcellular structures can be imaged directly in plunge-frozen adherent cells, in practice it is restricted to the cell periphery. In the study of HSV1 entry, it limited the number of captured entry events. To overcome this sample-inherent limitation, entry of HSV1 into synaptosome (physiologically active endings of neurons) was analysed [24] (Figure 3a). Synaptosomes are substantially thinner and enabled data acquisition with improved signal-to-noise ratio (SNR). Their round shape also allowed observing viral entry events in side views thus providing optimal orientation in respect to the missing wedge [35]. Snapshots of different membrane fusion steps including initial viral attachment, fusion pore formation and membrane dilation post-fusion could be captured [24]. Importantly, the experiments revealed that the proximal and less glycoprotein-studded pole of the virion is functionally the entry pole. Virus entry into liposomes is the next level of investigation system reduction. An example for this is shown in the sequence in Figure 3a and was extensively used for other viruses, e.g., influenza virus [36] and the retrovirus ASLV [37].

HSV1 entry, unlike many other viruses, involves the interaction of four viral glycoproteins, namely glycoproteins D, B, H and L, and at least one cellular receptor [38–40]. Individual glycoproteins were detected at the membrane fusion intermediate sites described above but the complexity of HSV1 and the limited resolution precluded confident assignment. In cases where hundreds or even thousands of identical copies of a macromolecule appear in a tomogram, sub-volumes containing the macromolecule can be extracted and averaged following an iterative process of orientation and positional refinement. This results in a 3D reconstruction of the macromolecule with improved SNR and resolution (for review see [41]). This so-called sub-volume averaging was recently applied to gB, a key component of the complex herpesvirus fusion machinery, and yielded a 3D reconstruction of gB bound to its target membrane [42] (Figure 3b). The resolution of this reconstruction allowed the fitting of the gB crystal structure. The EM reconstruction, together with the fitting, revealed that interaction of gB with target membrane was mediated by the fusion loops, and limited to the outer membrane leaflet. Applying a similar approach to other components of the herpesvirus fusion machinery will enhance our understanding of the complex mechanism of HSV fusion.

## HSV1 capsid assembly and nuclear egress

Herpesvirus capsids are assembled in the nuclei of infected cells. This poses a challenge: the ∼125-nm-diameter capsids are too large to pass the nuclear pore. The generally accepted model for crossing the nuclear envelope (nuclear egress) is the primary envelopment/de-envelopment model (recently reviewed in [43]). According to this model capsids bud at the inner nuclear membrane into the perinuclear cleft thus acquiring an envelope membrane. This primary envelope is then fused with the outer nuclear membrane leading to capsid release into the cytosol. Primary envelope formation is driven by the nuclear egress complex (NEC), a heterodimeric complex of two conserved HSV1 proteins, pUL34 and pUL31.

**Figure 4:**
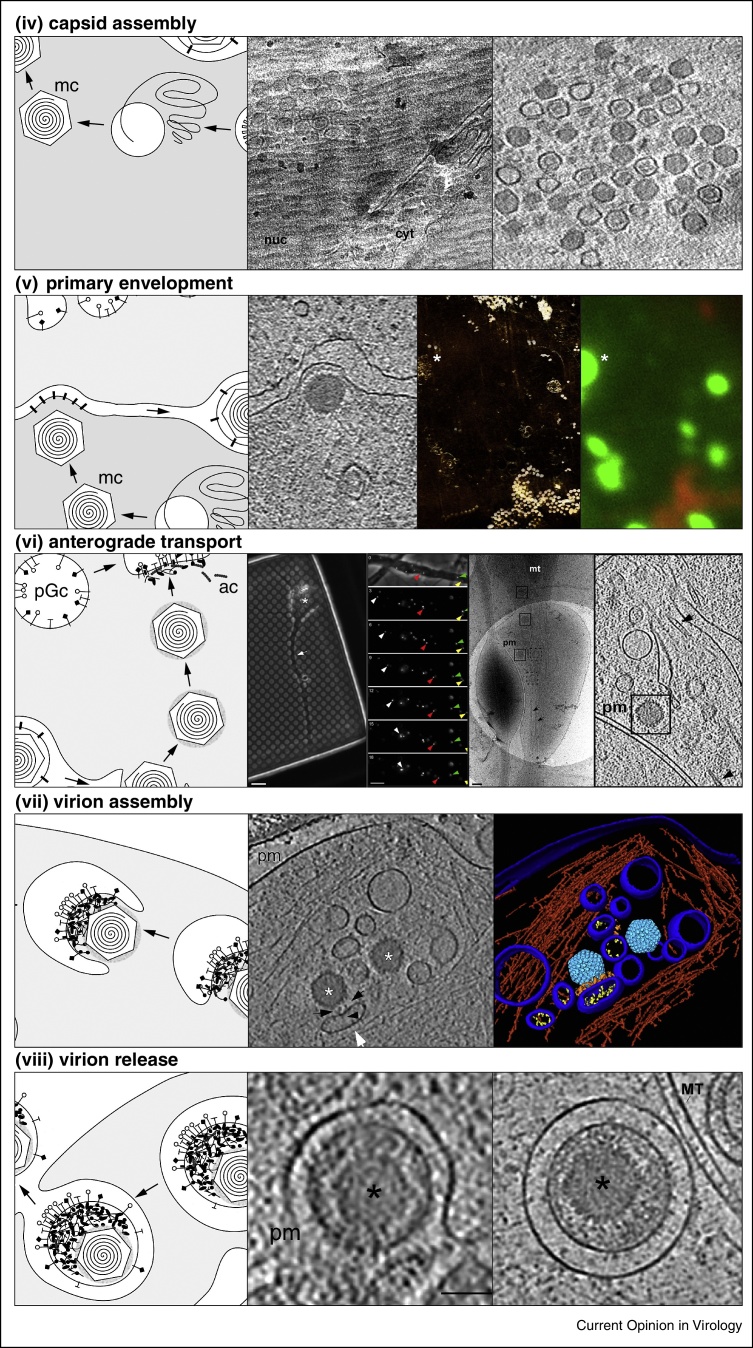
Dedicated experimental systems used for the study of herpesvirus assembly and transport *in vivo*. ***Capsid assembly***, electron cryo microscopy of vitreous sections (CEMOVIS) of mammalian cells infected with HSV1 (labelled with GFP) revealed the three distinct types of nucleocapsids close to the inner nuclear membrane (INM) [44]. Shown are a projection image (left) and a slice from a tomogram (right). ***Primary envelopment***, CEMOVIS of mammalian cell infected with murine cytomegalovirus provided snapshots of capsid primary envelopment at the INM (left panel), a layer of density most likely of the nuclear egress complex (NEC) was observed between the capsid and the INM [30]. Correlated imaging with fluorescence and soft X-ray microscopy of cells co-expressing both components of the NEC showed that the NEC is sufficient to drive formation of correctly sized primary envelopes [14]. ***Anterograde transport***, HSV1 infected hippocampal neurons grown directly on holey carbon EM grids were first analysed with live-cell fluorescence imaging that was then correlated with cryoEM and cryoET [48]. Capsids without envelopes were observed undergoing transport along the axon. ***Virion assembly***, secondary envelopment events were observed at axonal terminals [48,49]. Capsids budded into cellular vesicles that clearly were showing viral glycoproteins on their lumenal side and tegument proteins on the membrane side facing the capsid. Shown are a slice from a tomogram (left panel of the cryoEM panels) and 3D rendering of the tomogram (right panel). ***Virion release***, virions transported inside cellular vesicles (right panel) that then fuse at the plasma membrane (left panel) were observed at axon terminals by cryoET [49].

Electron cryo microscopy of vitreous sections (CEMOVIS) is a method of making specimens that are too thick for intact imaging (like the cell nucleus) amenable to cryoEM [11]. The specimens are first vitrified by high-pressure freezing, then sliced into thin sections that can subsequently be analysed by cryoEM or cryoET. Applying this technique in combination with fluorescence microscopy revealed capsid aggregates near the inner nuclear membrane [44] (Figure 4, first row). Three nucleocapsid types could be clearly distinguished, namely, empty A-capsids, scaffold protein containing B-capsids, and DNA-packed C-capsids. However, certain limitations have prevented CEMOVIS from becoming a more commonly used approach [45^•^]. A recently emerging alternative technique for sample thinning is targeted focused ion beam (FIB) milling (typically using gallium ions) for targeted abrasion of cryo specimens [46^••^,47].

An other alternative approach for imaging native cryo specimens that are too thick for cryoEM/ET is soft X-ray cryo microscopy. In this technique, data are collected within the ‘water window’ wavelength that provides images with high SNR and 3D resolution of currently ∼30 nm [15^•^]. Soft X-rays penetrate biological samples with thicknesses in the micrometre range. Correlated imaging using in-column cryo epi-fluorescence and soft X-ray microscopy of cells co-overexpressing the alphaherpesvirus NEC components showed that pUL31and pUL34-GFP are sufficient to drive membrane modulations that lead to virus-induced vesicular structures in the nucleus, expanding the nucleoplasmic reticulum [14] (Figure 4). The applied combination of cryoEM/ET with fluorescence imaging and soft X-ray cryo microscopy/tomography enabled a multiscale approach of unperturbed structures of interest.

## HSV1 virion assembly in primary neurons

HSV1 establishes lifelong latent infections in the peripheral nervous system. It is assumed that the processes of viral entry, transport to the cell body, capsid formation in the nucleus and nuclear egress are overall similar for neuronal and non-neuronal cells. The site of tegumentation and details of the secondary envelopment, however, remained debated. Using fluorescence live-cell imaging in correlation with cryoET addressed this open question [48]. For correlating live-cell fluorescence imaging with cryoEM, hippocampal neurons were grown directly on holey carbon EM grids (Figure 4). Sixteen hours post-infection, fluorescently labelled viral particles were observed undergoing mostly anterograde transport. CryoET data collected along the axon revealed that most transported particles were capsids without envelope. Secondary envelopment events were observed at axonal terminals [48,49] with capsids budding into vesicles clearly containing viral glycoprotein spikes on the vesicle lumen side of the membrane and tegument proteins on the membrane side facing the capsid. Filamentous actin surrounded the secondary envelopment sites in the axon terminal forming an assembly compartment [48].

## Outlook and prospects

Here we provided an overview of the application of the recently developed extensive ‘cryo tool-kit’ that enables studying even highly complex viral ‘life’ cycles as those of the herpesviruses in close-to-native conditions (Figure 2). This hybrid approach combines dynamic, live-cell imaging with static imaging of cryo-immobilized samples, and spans a resolution range from micrometres to angstroms (Figure 1). CryoEM and cryoET have emerged as prominent techniques to study virus–host interactions. They can be used to image cellular processes in the native state and can also provide *in situ* structures of macromolecular complexes at nanometer resolution. The introduction of direct electron detectors [50^••^] is currently revolutionizing the field and will likely allow atomic resolution reconstruction, possibly competing with or, ideally, matching X-ray crystallography [51^•^]. Routine application of phase plates in cryoET is another development that will have an impact on cellular tomography, and exciting first results imaging phages that assemble in their cyanobacterial hosts have recently been reported [52^••^]. The inherent limitation for the application of cellular cryoET is the thickness of the sample. Recent reported results with FIB milling under cryogenic conditions suggest that it will become the method of choice for sample thinning for cryoET [46^••^,53]. A proof of principle experiment based on serial slicing of frozen-hydrated cells and even tissues using a FIB combined with SEM block-face imaging has recently been demonstrated to produce images with a lateral resolution of a few nanometers and slice thicknesses of 30 nm [54^•^]. Ongoing effort is put on accurate correlation between advanced light/fluorescence microscopy and cryoEM [55], and the introduction of cryo-fluorescence microscopy now allows for more precise correlations [56^•^,57]. Thus, an integrated approach of high-resolution structure determination methods and correlative light, soft X-ray and EM allows us to look at dynamic biological processes at different resolutions and complexity.

Applying this ‘cryo tool-kit’ in studying virus–host interactions will undoubtedly lead to a better perception of the complexity of the underlying processes and enable us to unveil novel cellular mechanisms and pathways.

## References and recommended reading

Papers of particular interest, published within the period of review, have been highlighted as:

• of special interest
•• of outstanding interest
